# How animals discriminate between stimulus magnitudes: a meta-analysis

**DOI:** 10.1093/beheco/araf025

**Published:** 2025-03-24

**Authors:** Megan Z Worsley, Julia Schroeder, Tanmay Dixit

**Affiliations:** Department of Life Sciences, Imperial College London, Buckhurst Road, Ascot, SL57PY, United Kingdom; Department of Life Sciences, Imperial College London, Buckhurst Road, Ascot, SL57PY, United Kingdom; Department of Zoology, University of Cambridge, Downing Street, Cambridge CB23EJ, United Kingdom; FitzPatrick Institute of African Ornithology, Department of Biological Sciences, University of Cape Town, Rondebosch, Cape Town, Private Bag X3 Rondebosch 7701, South Africa

**Keywords:** Weber's law, proportional processing, meta-analysis, receiver perception, sensory systems, magnitude effect

## Abstract

To maximize their fitness, animals must often discriminate between stimuli differing in magnitude (such as size, intensity, or number). Weber’s Law of proportional processing states that stimuli are compared based on the proportional difference in magnitude, rather than the absolute difference. Weber’s Law implies that when stimulus magnitudes are higher, it becomes harder to discriminate small differences between stimuli, leading to more discrimination errors. More generally, we can refer to a correlation between stimulus magnitude and discrimination error frequency as a magnitude effect, with Weber’s law being a special case of the magnitude effect. However, the strength and prevalence of the magnitude effect across species have never previously been examined. Here, we conducted a meta-analysis to quantify the strength of the magnitude effect across studies, finding that, on average, perception followed Weber’s Law. However, the strength of the magnitude effect varied widely, and this variation was not explained by any biological or methodological differences between studies that we examined. Our findings suggest that although its strength varies considerably, the magnitude effect is commonplace, and this sensory bias is therefore likely to affect signal evolution across diverse systems. Better discrimination at lower magnitudes might result in signalers evolving lower magnitude signals when being discriminated is beneficial, and higher magnitude signals when being discriminated is costly. Furthermore, selection for higher magnitude signals (eg sexual ornaments) may be weakened, because receivers are less able to discriminate as signal magnitudes increase.

## Teaser

Often, as the magnitude of a stimulus increases, it becomes harder to detect small changes in this stimulus (termed the magnitude effect). We investigated how commonly this effect applies across a range of species and stimuli. We found that on average, there is a magnitude effect, but that the strength of this effect varies a lot between species. The magnitude effect can affect animal decision-making and the evolution of signals.

## Introduction

To exhibit adaptive behavior, animals must make informed decisions (Dall et al. 2005). However, to make informed decisions, animals must accurately assess their environment (Stevens 2013). Sensory systems cannot perfectly capture all the information contained in the environment (Laughlin 2001; Niven and Laughlin 2008), and much of the information animals do receive is not relevant to their fitness (Lev-Ari et al. 2022; Tibbetts et al. 2024), so sensory systems are biased to extract the most important information efficiently. Such biases can affect decision-making (Endler 1992; Endler and Basolo 1998), so to understand behavior, we must also understand the common biases of sensory systems (Stevens 2013).

### Weber’s law

**Fig. 1.**
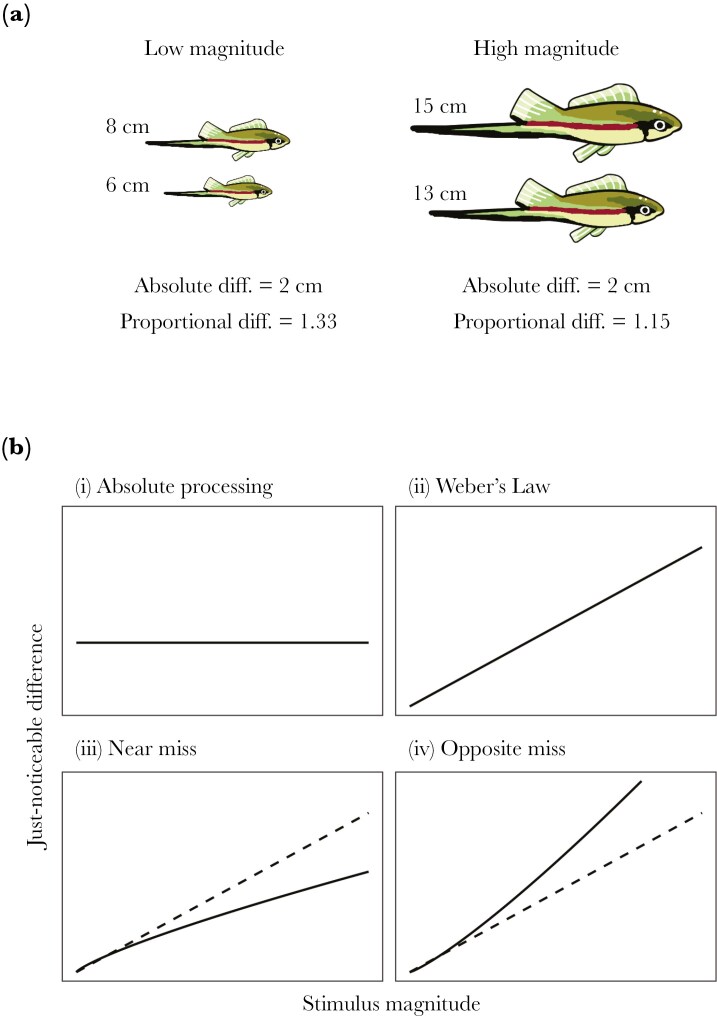
The magnitude effect. (A) Humans perceive length according to Weber’s Law. The magnitude (length) of the left pair of fish is smaller than the right pair of fish, so although the absolute difference in length between the fish is the same (2 cm), it is easier to detect the larger fish in the pair on the left, because the proportional size difference is larger. (B) How the just-noticeable difference varies with stimulus magnitude, under different models of perception. (i) If only the absolute difference contributes to discrimination, the just-noticeable difference is constant, regardless of stimulus magnitude. (ii) Under Weber’s Law, the just-noticeable difference is proportional to stimulus magnitude. (iii) Under the near miss to Weber’s Law (solid line), the just-noticeable difference increases with stimulus magnitude, but less than expected under Weber’s Law (dashed line). (iv) Under the opposite miss to Weber’s Law (solid line), the just-noticeable difference increases with stimulus magnitude even more than expected under Weber’s Law (dashed line).

Animals often make decisions by comparing the magnitudes of two stimuli. For example, stimuli might aid animals in choosing between patches containing different amounts of food, or between mates with ornaments of different sizes (Bullough et al. 2023). This comparison often follows Weber’s Law, which states that stimuli are compared not based on the absolute difference between them, but rather the proportional difference (ie the difference relative to the stimulus magnitude; Weber 1834; Fechner 1966; Bullough et al. 2023). Under Weber’s Law, as the magnitude of a stimulus increases, it becomes more difficult to detect small differences in magnitude (Cohen 1984) (Fig. 1A), leading to more discrimination errors.

If the stimulus which is perceived according to Weber’s Law is a signal, meaning a stimulus which has evolved to alter the behavior of a receiver (Krebs and Dawkins 1984), Weber’s law can affect the evolution of this signal (Akre and Johnsen 2014). For example, many male sexual signals evolve via sexual selection, with females mating preferentially with the males with higher magnitude signals. This means that there is selection for higher magnitude signals, and the signal magnitude is expected to increase over evolutionary time (Fisher 1930). However, Weber’s law means that as the magnitude of this sexual signal increases, it becomes more difficult for females to discriminate between males with similar signals. This could have several possible evolutionary consequences. One possibility is that as signal magnitude increases, the selection pressure for higher magnitude signals may weaken (Akre and Johnsen 2014; Nachev et al. 2017). An alternative possibility is that as signal magnitude increases, signals would need to have higher and higher magnitudes for the difference to be detectable, so the rate at which signals evolve higher magnitudes may escalate over time (Akre and Johnsen 2014). These possibilities, and other potential implications of Weber’s law, are reviewed in Akre and Johnsen (2014). Weber’s Law has also been suggested to affect the evolution of other types of signals, such as the rewards used by flowers to attract pollinators (Nachev et al. 2017), and signals that are subject to mimicry (Dixit et al. 2021).

Weber’s Law has been demonstrated widely, for many different stimuli and in many different species (reviewed in Akre and Johnsen 2014). Stimuli that are perceived according to Weber’s Law are used in a variety of ecological contexts, such as foraging (Akre et al. 2011; Nachev et al. 2013, 2017), mate choice (Cohen 1984; Akre et al. 2011; Ryan et al. 2019; LaBarbera et al. 2020; Caves and Kelley 2023), group cohesion (Agrillo et al. 2008; Gómez-Laplaza and Gerlai 2011; Perna et al. 2012), and mimicry (Dixit et al. 2022). Since this sensory bias is found in a variety of species and contexts, it potentially has far-reaching evolutionary consequences (Akre and Johnsen 2014).

### Near miss and opposite miss to weber’s law

Weber’s Law states that the just-noticeable difference—the smallest increment or difference that can be perceived—is not a constant threshold (Fig. 1B(i)), but increases linearly with the magnitude of the stimulus (Fig. 1B(ii)) (Weber 1834; Fechner 1966). However, many studies have found that the effect of stimulus magnitude on discrimination ability is either weaker or stronger than predicted by Weber’s Law. Therefore, Weber’s Law can be described as a special case of a more general magnitude effect (Nachev et al. 2013). The near miss to Weber’s Law describes a relationship where discrimination becomes less accurate as stimulus magnitudes increase, but this effect is less strong than Weber’s Law predicts (McGill and Goldberg 1968) (Fig. 1B(iii)). The near miss to Weber’s Law has been demonstrated in sound intensity discrimination in birds and mammals (Forrest 1994). Conversely, the opposite miss to Weber’s Law describes a relationship where discrimination becomes even less accurate at higher stimulus magnitudes than Weber’s Law predicts (Forrest 1994) (Fig. 1B(iv)). The opposite miss to Weber’s Law has been demonstrated in sound intensity discrimination in crickets and frogs (Forrest 1994), sugar concentration discrimination in bats (Nachev et al. 2013), size discrimination in green swordtails (Caves and Kelley 2023), and duration (LaBarbera et al. 2020) and sound intensity (Bee et al. 2012) discrimination in treefrogs. Furthermore, while the magnitude effect has been shown in a range of systems, it is not universal. In bumblebees and honeybees, for example, discrimination between two concentrations of sugar depends only on the absolute difference in concentration (Nachev et al. 2013) (Fig. 1B(i)).

The variation between these previous studies suggests that the strength of the magnitude effect varies across studies, species, and stimuli. This in turn means that different signals may have different evolutionary trajectories. For example, under Weber’s Law, the benefits of evolving higher magnitude signals are predicted to decrease with stimulus magnitude; under the opposite miss to Weber’s Law, these benefits would decrease even more sharply (Nachev et al. 2013; LaBarbera et al. 2020; Dixit et al. 2021); under the near miss, the benefits would decrease, but less sharply than under Weber’s Law (Dixit et al. 2021). Therefore, for evolutionary biologists or ecologists wishing to study if and how individuals make optimal decisions, or to predict how signals will evolve, it is important to confirm whether the magnitude effect is present, and the strength of this effect.

While several studies have tested for Weber’s Law or the magnitude effect more generally, few have quantified the strength of this effect, particularly in an ecological context (but see Nachev et al. 2013; Dixit et al. 2022; Caves and Kelley 2023). In this study, we performed a meta-analysis to quantify the magnitude effect across a wide range of existing studies. We aimed to answer three questions: (1) How common is Weber’s Law? (2) When perception diverges from Weber’s Law, are there any trends in the direction and degree of this divergence? And (3) Does the magnitude effect vary across species and sensory modalities, and/or with experimental design?

## Methods

### Literature search

First, we performed a literature search to find experimental studies in which animals had to discriminate between stimuli of different magnitudes. The search string was iteratively refined to optimize the number of relevant results. In particular, search terms usually associated with studies on human participants were excluded, to focus on ecologically relevant decision-making.

The final search was conducted on December 2^nd^ 2024 in two databases, Web of Science and Scopus (Grames et al. 2019), for studies published after 1990. The final search strings were:

Web of Science: ALL = ((psychophysic* OR (sensory AND perce*) OR “Weber* Law”) AND ((discrimin* OR prefer*) AND stimul*) NOT (human* OR observer* OR participant* OR patient* OR listen* OR child* OR men OR women OR people)) NOT DT = (Review)

Scopus: ALL ((psychophysic* OR “Weber* Law”) AND ((discrimin* OR prefer*) AND stimul*) AND NOT (human* OR observer* OR participant* OR patient* OR listen* OR child* OR men OR women OR people)) AND SUBJAREA(agri OR bioc OR neur OR psyc) AND NOT DOCTYPE(re)

The search returned 1,852 results from Web of Science and 1,999 results from Scopus. We also screened a table of studies in Akre and Johnsen (2014), a review on the prevalence of Weber’s Law, which contained 39 references. Studies from the different sources were combined, and duplicates were removed using litsearchr (version 1.0.0, Grames et al. 2019). In total, 3780 studies were screened.

### Study selection and data acquisition

The search results were manually screened to find relevant studies. Studies were included if they met all the following criteria: (1) the subjects were non-human animals; (2) the study involved a behavioral choice test, in which stimuli were discriminated between; and (3) the magnitude of the stimuli was measured along a clear axis, for which stimulus magnitudes can be zero, but are always non-negative. This third criterion excludes some continuous stimuli such as hue and angle, because while an axis can potentially be defined, it is unclear whether the scale has a meaningful “zero” (Dixit et al. 2021).

**Fig. 2.**
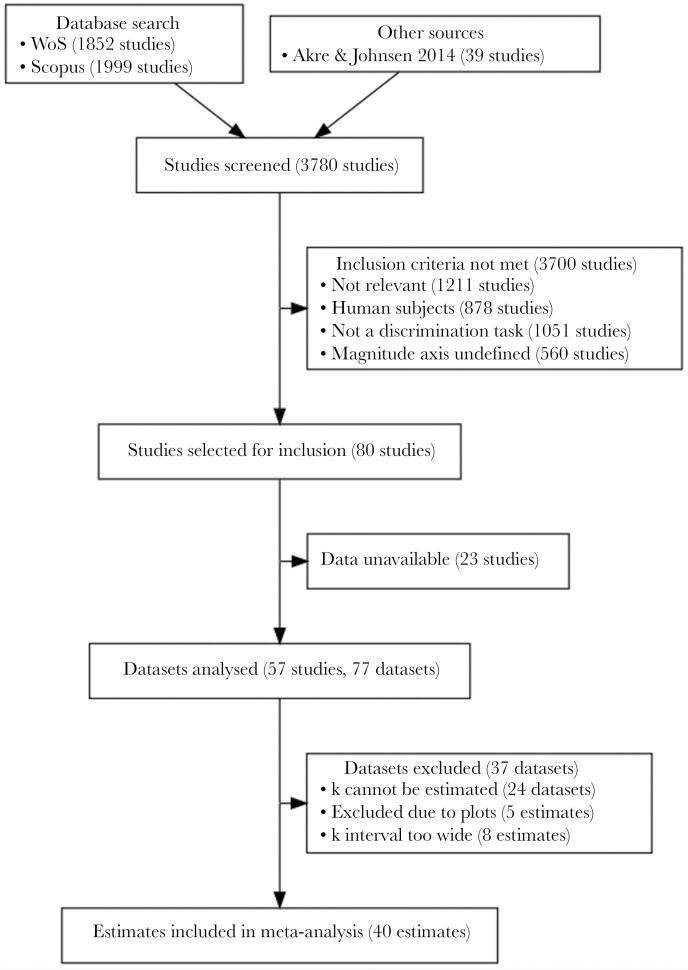
A flowchart of the literature search, screening, and data extraction process. Modified from the PRISMA recommended reporting (Moher et al. 2009). A list of the datasets analyzed can be found in Supplementary Data 1, and the full literature search can be found in Supplementary Data 2.

Where necessary, data were extracted from figures using GraphGrabber v2.0.2 (Benbow 2020), or the authors were contacted to provide additional data. Of the 80 studies shortlisted for analysis, there were 23 studies for which data could not be obtained (Fig. 2). From the remaining 57 studies, 77 datasets were obtained, due to multiple different experiments being conducted in some studies.

### Quantifying the magnitude effect

All analysis and visualization was conducted using R 4.2.2 (R Core Team 2022) and *tidyverse* (Wickham et al. 2019). Following Dixit et al. (2022) and Nachev et al. (2013), the relationship between the magnitudes of a pair of stimuli and discrimination performance was modeled with a generalized form of the equation describing Weber’s Law, which includes a parameter (*k*) for the strength of the magnitude effect. In this model, the perceived difference between two stimuli is defined as:


$$\begin{array}{l} perceived\;difference=\;\frac{\Delta I}{I^{k}} \end{array}$$

Equation 1

where ΔI is the absolute difference between the magnitudes of the two stimuli, I is the overall stimulus magnitude, and *k* is a constant which determines how much I impacts the perceived difference. Since there are two different stimuli in the pair, stimulus magnitude I (ie the denominator) is poorly defined, and several different definitions have previously been used (eg Nachev et al. 2013; Dixit et al. 2022; Caves and Kelley 2023). Following Nachev et al. (2013), we defined I as the arithmetic mean of the two stimulus magnitudes in each pair, as this definition can be consistently applied across studies.

There are two important special cases of Equation 1: *k *= 0, and *k *= 1 (Dixit et al. 2022). When *k *= 0, discrimination depends only on the absolute difference ΔI between the two stimulus magnitudes:


$$\begin{array}{l} perceived\;difference\;=\;\frac{\Delta I}{I^{0\;}}=\;\Delta I \end{array}$$

Therefore, if *k *= 0, there is no magnitude effect (Fig. 1b(i)).

When *k *= 1, Equation 1 reduces to:


$$\begin{array}{l} perceived\;difference\;=\;\frac{\Delta I}{I^{1\;}}=\;\frac{\Delta I}{I^{\;}} \end{array}$$

which is equivalent to Weber’s Law (Fechner 1966). Therefore, if *k *= 1, then the magnitude effect is exactly as strong as Weber’s Law predicts (Fig. 1b(ii)).

The near miss (Fig. 1b(iii)) and opposite miss (Fig. 1b(iv)) scenarios can also be described by Equation 1, with 0 <* k *< 1 and *k *> 1 respectively. Therefore, the value of *k* that best predicts discrimination performance is a measure of the strength of the magnitude effect (Nachev et al. 2013; Dixit et al. 2022).

### Estimating the strength of the magnitude effect

We created a custom R package, *kber* (Worsley and Dixit 2024), to estimate the value of *k* that best predicts discrimination performance in each study. Generalized linear models were used to predict how the perceived difference between two stimuli affects discrimination performance. Following the definition of perceived difference from Equation 1, these models were of the form:


$$\begin{array}{l} discrimination\;performance\;∼\;\frac{\Delta I}{I^{k}} \end{array}$$

Equation 2

The measure of discrimination performance, and hence the specifics of this model, varied between studies. Discrimination performance was usually provided as a proportion of correct choices, in which case a binomial logistic regression was used. However, some studies provided discrimination performance as a continuous preference index, in which case a linear regression was used.

For each dataset, we used the function *estimate_k* from *kber* (Worsley and Dixit 2024) to estimate the best-fitting value of *k*, defined as the value which minimizes the AIC of the model given in Equation 2. This function also provides 95% confidence intervals, defined as the range of values of *k* for which the resulting model has an AIC within 2 units of the minimum AIC. Furthermore, to enable easy troubleshooting, *estimate_k* produces diagnostic plots showing the relationship between *k* and the resulting model AIC. For some datasets (n = 24), the function could not converge on an estimate for *k*, and we used these diagnostic plots to confirm that estimating *k* was not possible. We also used these plots to identify estimates which may be unreliable (for example, if the plot has multiple local minima, corresponding to multiple possible optimal values of k), and we excluded such estimates from the meta-analysis (n = 4). Furthermore, we plotted the fitted model against the data, and excluded datasets which showed clear overfitting (n = 1). A full list of excluded datasets and their reasons for exclusion is available in Supplementary Data 1.

We used the 95% confidence interval for each *k* estimate to determine whether a magnitude effect was present, and if so, whether the strength of this effect follows Weber’s Law. If *k *= 0 (which corresponds to no magnitude effect) falls outside of the 95% confidence interval, then stimulus magnitude has a significant effect on discrimination. Likewise, if *k *= 1 (which corresponds to Weber’s Law) falls outside of the 95% confidence interval, then discrimination significantly diverges from Weber’s Law (Dixit et al. 2022).

To avoid including low-quality estimates of *k*, we excluded estimates for which the difference between the upper and lower confidence intervals for *k* was greater than 3 (n = 8). In total, 40 *k* estimates were obtained (Supplementary Data 1). 28 estimates were from the literature search, 10 were from Akre and Johnsen (2014), and two were found in both the search and Akre and Johnsen (2014). The lack of overlap between the database search and the review suggests that the database search missed relevant studies (see discussion).

### Statistical analysis

All meta-analysis was conducted using the package *metafor* 3.8-1 (Viechtbauer, 2010). We used the estimated value of *k* as the effect size for each study. The standard error, calculated from the 95% confidence interval (Higgins et al. 2023), was used to weight each estimate of *k*.

To estimate the global mean value of *k*, a linear phylogenetic mixed-effects model with no fixed effects was fitted with the function *rma.mv* from *metafor*. Random effects for study (to account for multiple datasets published in the same paper), species, and phylogeny were included, to account for potential correlations between *k* estimates due to biological or methodological similarities (Hadfield & Nakagawa, 2010). Phylogeny was included as a random effect by obtaining a matrix of expected phylogenetic correlations between each pair of species (Lynch 1991) using the package *ape* (Paradis and Schliep 2019), with branch lengths estimated from the topology of the phylogenetic tree (Pagel 1999; Horváth et al. 2023). The effect of including each of these random effects was determined using likelihood ratio tests, removing each random effect in turn. I^2^ was used as a measure of the heterogeneity of the *k* estimates (Higgins & Thompson, 2002), and this was calculated from the mixed-effects model using *orchaRd* (Nakagawa et al. 2023) following Nakagawa & Santos (2012).

We also tested whether variation in the value of *k* was predicted by four different fixed effects: sensory modality, the type of discrimination task, whether the choices were conditioned or innate, and whether the study considered Weber’s Law when interpreting its results (Table 1). These variables were all included in a single model along with the random effects, to determine whether any of the fixed effects significantly affect *k*. To test for collinearity, chi-squared tests were conducted between each pair of fixed effects, and generalized variance inflation factors (GVIFs) were calculated using the *vif.rma* function. These GVIFs were compared to simulated distributions of the values expected if the variances were independent using the ‘*sim*’ argument of the *vif.rma* function in *metafor* (Viechtbauer 2010).

**Table 1.** Structure of the fixed effects included in the meta-analysis.

Moderator	Description	Levels
Sensory modality	The sensory modality of the stimulus	Electroreception, hearing, smell, taste, touch, vision
Task type	Whether the discrimination task involved ranking two stimuli by their magnitudes, or simply detecting that the two stimuli are different	Rank, difference
Choice type	Whether the response to the chosen stimulus was conditioned or innate	Conditioned, innate
Weber’s Law	Whether the study referenced Weber’s Law when interpreting its findings	Reported, not reported

We tested for two trends that would suggest publication bias: (1) studies with smaller sample sizes showing larger effect sizes, and (2) effect sizes decreasing over time (Nakagawa and Santos 2012). To test the former, we performed an Egger’s regression test (Egger et al. 1997), using the standard error as the predictor variable and including the same random effects as above. We also used a funnel plot to visualize the relationship between these variables; an asymmetrical funnel plot is evidence for an association between precision and effect size. To test whether effect sizes tend to decrease over time, we performed a second Egger’s regression test using publication year as the predictor variable.

## Results

In total, 40 *k* estimates from 34 studies were included in the meta-analysis, with 29 species represented, ranging from roundworms and insects to frogs, birds, and mammals. Perceived difference (computed from Equation 1 using the estimated value of k) significantly predicted discrimination for all included datasets (Supplementary Data 1). For 34 of the 40 *k* estimates, *k* differed significantly from zero. This shows that discrimination performance varied significantly with stimulus magnitude, which is evidence for a magnitude effect. However, the direction of this effect was highly variable. 13 datasets approximately followed Weber’s Law (*k *= 1), 8 datasets showed the “near miss” to Weber’s Law (0 <* k *< 1), and 10 datasets showed the “opposite miss” to Weber’s Law (*k* > 1). Most surprisingly, 3 datasets showed a magnitude effect in the opposite direction to Weber’s Law (*k* < 0)—in other words, as stimulus magnitude increased, discrimination performance also increased. Overall, most datasets showed evidence of a magnitude effect, but the strength and direction of this effect was highly variable.

**Fig. 3.**
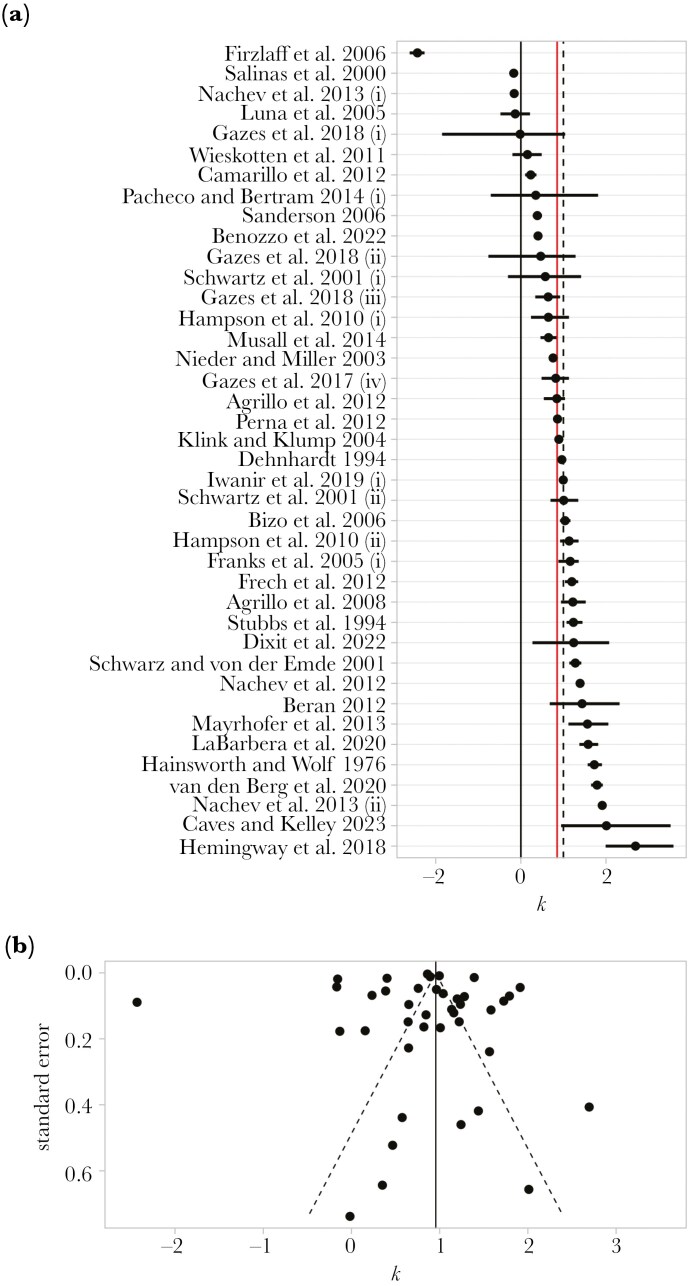
Distribution of estimates of the magnitude effect. (A) A forest plot of *k* estimates from each study, with horizontal bars showing the 95% confidence intervals for these estimates. References are shown on the left, with bracketed roman numerals differentiating separate estimates from the same study (see Supplementary Data 1 for further details about each study). Vertical reference lines show *k *= 0 (dashed black line), which corresponds to absolute processing, and *k *= 1 (solid black line), which corresponds to Weber’s Law. The red vertical line shows the meta-analytic mean *k* estimate (*k *= 0.96, 95% CI = 0.62–1.29). (B) A funnel plot showing the standard error of each study against the estimated value of *k*. The solid vertical line shows the meta-analytic mean *k* estimate (*k *= 0.96), and dashed diagonal lines show the 95% confidence intervals for this estimate (95% CI = 0.62–1.29). Egger’s regression test showed no significant funnel plot asymmetry (estimate = -0.54 ± 0.62, Z = -0.87, p = 0.384). Data from Hainsworth and Wolf (1976), Dehnhardt (1994), Stubbs et al. (1994), Salinas et al. (2000), Schwartz et al. (2001), Schwarz and von der Emde (2001), Nieder and Miller (2003), Klink and Klump (2004), Franks et al. (2005), Luna et al. (2005), Bizo et al. (2006), Firzlaff et al. (2006), Sanderson (2006), Agrillo et al. (2008), Hampson et al. (2010), Wieskotten et al. (2011), Agrillo et al. (2012), Beran (2012), Camarillo et al. (2012), Frech et al. (2012), Nachev and Winter (2012), Perna et al. (2012), Mayrhofer et al. (2013), Nachev et al. (2013), Musall et al. (2014), Pacheco and Bertram (2014), Gazes et al. (2018), Hemingway et al. (2018), Iwanir et al. (2019), LaBarbera et al. (2020), van den Berg et al. (2020), Benozzo et al. (2022), Dixit et al. (2022), Caves and Kelley (2023).

**Fig. 4.**
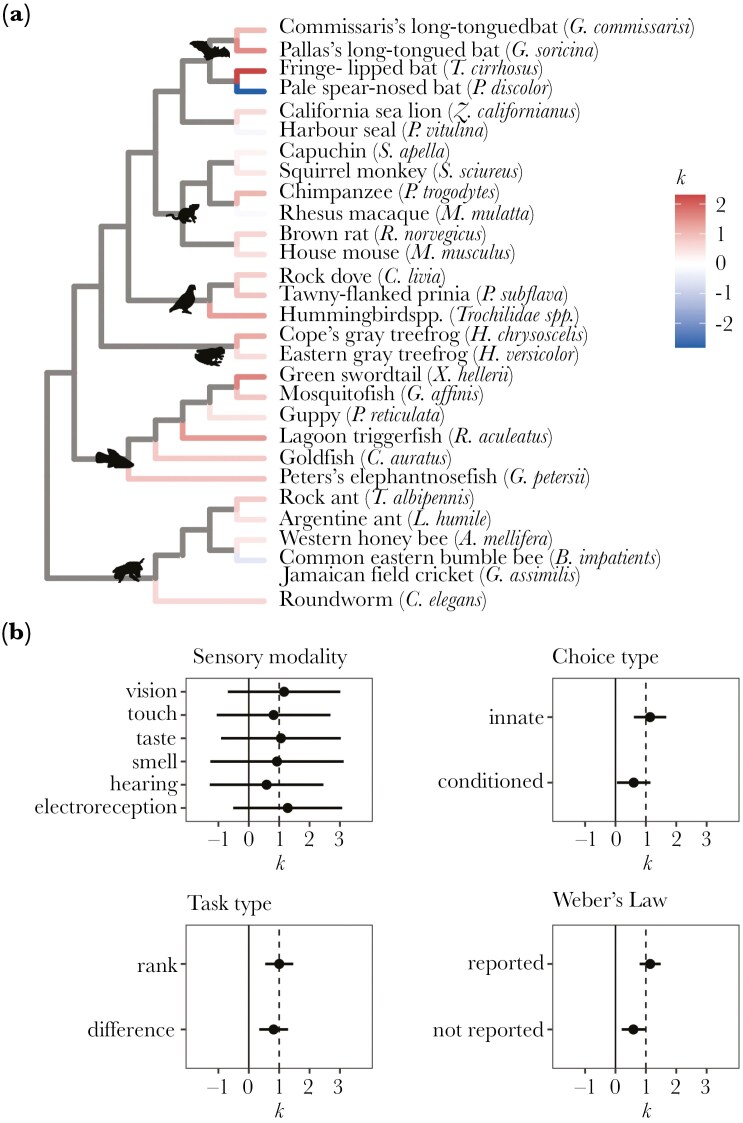
(A) How *k* varies across the phylogenetic tree. While variation in species explains some of the variation in *k*, phylogeny explains almost none of this variation (Table 2). This can be seen in this figure: *k* varies across the tree, but the *k* estimates of closely related species are not strongly correlated. Full species names are provided in Supplementary Data 1. (B) How the average value of *k* varies with each moderator. Vertical reference lines show *k *= 0 (solid black line), which corresponds to absolute processing, and *k *= 1 (dashed black line), which corresponds to Weber’s Law. The points show the estimated mean value of *k* for each group, and the error bars show the 95% confidence intervals. These values were estimated using single-variable meta-regressions for each fixed effect, accounting for variation in study (the study from which each dataset was extracted), species (the species being tested), and phylogeny (the phylogenetic relatedness between these species). In a full model containing all fixed effects, none of these fixed effects significantly affected *k* (Table 2).

A meta-regression with no random effects found an overall *k* estimate of 0.85 (95% CI = 0.60–1.11). This suggests that on average, discrimination approximately follows Weber’s Law (*k *= 1). However, the heterogeneity of *k* values in this model was extremely high, with an I^2^ value of 99.89%. Adding random effects to account for variance due to study, species, and phylogenetic effects, we found an overall *k* estimate of 0.96 (95% CI = 0.62–1.29) (Fig. 3A). Study, species, and phylogeny only explained a small amount of the variance in *k*, compared to the residual variance (Table 2). Likelihood ratio tests showed that the amount of variance explained by study and species effects was significantly greater than zero, but phylogeny did not explain a significant amount of variation (Table 2, Fig. 4A). The proportion of the total variance that can be explained by phylogeny is equivalent to Pagel’s λ (Pagel 1999; Horváth et al. 2023); accordingly, we found no phylogenetic signal for *k* (*λ* < 0.001, *p* = 1.000). Despite this, all three random effects were retained in the final model, as removing the phylogenetic component of variance can cause bias (Cinar et al. 2022).

**Table 2.** The amount of variance in the value of *k* explained in the meta-regression model. σ^2^ = the amount of variance explained by each variable; n levels = the number of levels for each variable; LRT = the test statistic output from a likelihood ratio test removing each variable in turn; p-value = the p-value of this likelihood ratio test.

Random effects	σ^2^	% variance explained	n levels	LRT	p-value
Study	0.1211	0.4%	34	232.7	< 0.0001
Phylogeny	0.0000	0.0%	29	0	1.000
Species	0.6586	2.4%	29	15.07	< 0.0001
Residual variance	27.01	97.2%	40		

### Publication bias

The funnel plot was largely symmetrical (Fig. 3B), and an Egger’s regression test for outcome reporting bias found no evidence for an association between high effect size and low precision (estimate = -0.54 ± 0.62, *Z* = -0.87, *p* = 0.384), so there is little evidence for publication bias. An Egger’s regression test for time-lag bias also found no evidence that the effect size changed over time (estimate = 0.007 ± 0.01, *Z* = 0.55, *p* = 0.584).

### Differences between subgroups

We found that *k* did not significantly differ with the type of discrimination task, whether the preference for the stimulus was innate or conditioned, the sensory modality of the stimulus, or whether the study reported Weber’s Law (Table 3; Fig. 4B). The latter suggests that while some sources used in the literature searched were biased (such as Akre and Johnsen (2014), which explicitly searched for studies showing Weber’s Law), this bias is unlikely to have affected the results.

**Table 3.** Fixed effect estimates from a meta-regression of the value of k, with the standard error, 95% confidence interval, Z-value, and p-value of each estimate. The reference values for the intercept are: Choice type: conditioned; Task type: difference; Sensory modality: electroreception; Weber’s Law: reported. “.” denotes a p-value below *p* = 0.100.

Moderator	Estimate	SE	95% CI	Z-value	p-value
Intercept	1.73	0.94	−0.11–3.58	1.84	0.066.
Choice type: innate	0.55	0.32	−0.07–1.16	1.74	0.082.
Task type: rank	−0.06	0.23	−0.51–0.38	−0.29	0.775
Sensory modality: hearing	−1.20	0.96	−3.07–0.68	−1.25	0.212
Sensory modality: smell	−1.09	1.13	−3.31–1.12	−0.97	0.333
Sensory modality: taste	−1.00	1.03	−3.01–1.02	−0.97	0.332
Sensory modality: touch	−0.91	0.94	−2.76–0.94	−0.97	0.333
Sensory modality: vision	−0.82	0.97	−2.71–1.08	−0.84	0.398
Weber’s Law: not reported	−0.39	0.30	−0.99–0.21	−1.27	0.203

Chi-squared tests showed that Choice type (whether the choice between stimuli was innate or conditioned) was correlated with Sensory modality (Cramer’s *V* = 0.623, *p* = 0.008**) and Task type (whether the subject had to determine which magnitude was larger, or simply detect whether the stimuli were different) (Cramer’s *V* = 0.404, *p* = 0.011*), which may limit our ability to disentangle the effects of these variables on *k*. These correlations can be explained by differing trends in the studies measuring innate choices compared to studies measuring conditioned choices. Innate responses to stimuli were almost always associated with directional preferences, so studies measuring innate responses were much more likely to test the subject’s ability to rank stimuli by their magnitudes, rather than simply detecting differences. These stimuli were also more likely to be olfactory (eg chemoattractants) or gustatory (eg rewarding food). We included all fixed effects in the final model to reduce the chance of Type I errors due to omitted variable bias (Lipsey 2003; Cinar et al. 2021), and found that the generalized variance inflation factors (GVIFs) for Choice, Task, Modality, and Weber’s law were not extreme compared to the simulated distribution of expected values (Table 4), suggesting that multicollinearity is not a major problem in this final model.

**Table 4.** Estimates of the degree of multicollinearity for each variable. Cramer’s V and corresponding p-values are calculated from chi-squared tests between each pair of variables. “GVIF” gives the generalized variance inflation factor for each variable. “Proportion” gives the proportion of the distribution of simulated GVIF values that are lower than the actual GVIF value, with 0.5 signifying a GVIF value which is the median of the simulated distribution, and 1 signifying an extremely high GVIF value.

Variable	Correlations with other variables				no. levels	GVIF	Proportion
	Choice	Task	Modality	Weber’s law			
**Choice**		V = 0.623 (p = 0.008**)	V = 0.404 (p = 0.011*)	V = 0.153 (p = 0.333)	2	1.32	0.237
**Task**	V = 0.623 (p = 0.008**)		V = 0.354 (p = 0.413)	V = 0.059 (p = 0.709)	2	1.37	0.317
**Modality**	V = 0.404 (p = 0.011*)	V = 0.354 (p = 0.413)		V = 0.446 (p = 0.242)	6	2.82	0.551
**Weber’s law**	V = 0.153 (p = 0.333)	V = 0.442 (p = 0.167)	V = 0.059 (p = 0.709)		2	2.01	0.727

## Discussion

Stimulus magnitude significantly affected discrimination performance in most of the datasets we analyzed; in other words, there was evidence of a magnitude effect in most studies. On average, discrimination conformed approximately to Weber’s Law. However, individual estimates of the strength of the magnitude effect were highly variable, with perception often diverging from Weber’s Law. The extent and direction of this divergence was also highly variable, with examples of both the “near miss” and “opposite miss” to Weber’s Law, where magnitude affects discrimination less and more strongly than Weber’s Law respectively. There were also a small number of studies for which discrimination became easier at higher magnitudes: the opposite of Weber’s Law. In the set of studies we included, there was substantial variation in species, stimuli, methods, and aims, and it seems likely that this variation contributed to the high heterogeneity in the results. We found that study and species accounted for a small but significant amount of the variation in perception. However, while perception varied with species, phylogeny did not play a role in this variation; more closely related species did not show more similar perception than more distant species. Furthermore, the magnitude effect did not vary with the type of discrimination task (whether the subject had to determine which magnitude was higher or simply detect that the stimuli were different), whether the preference for the stimulus was innate or conditioned, the sensory modality of the stimulus (vision, touch, taste, smell, hearing, electroreception), or whether the study reported Weber’s Law. Therefore, the high heterogeneity between studies was largely unexplained.

Despite this heterogeneity, our results suggest that discrimination usually becomes harder as the magnitude of stimuli increases, and that on average, this effect follows Weber’s Law. When the stimuli being perceived are signals, this sensory bias will likely impose selection on signal magnitude, but the direction of this selection depends on the nature of the signal. In general, when the signaler benefits from their signal being discriminated, there should be selection for their signal to be more distinguishable from other signals (ie selection for lower stimulus magnitudes), and when being discriminated is costly, there should be selection for the signal to be less distinguishable (ie selection for higher stimulus magnitudes) (Dixit et al. 2021). This principle can be applied to systems in which receivers have a directional preference for higher signal magnitudes, such that signalers benefit from their signals being detectably higher magnitude than those of other signalers. This preference could be because signalers demonstrate their quality or fitness through signals such as sexual ornaments in males or honest signals to pollinators in flowers, or because signalers benefit from sensory biases in receivers favoring higher signal magnitudes (Ryan and Keddy-Hector 1992). However, the magnitude effect would result in diminishing benefits as signal magnitude increases (as receivers would be less able to detect incremental differences in these signals). This might cause the strength of selection for higher magnitude signals used in sexual selection to decrease over time (Akre and Johnsen 2014), or alternatively, it might select for larger incremental changes in signal magnitude so that they are detectable by the receiver (Akre and Johnsen 2014). The magnitude effect is also relevant in scenarios where there are no fixed directional preferences, and receivers are simply detecting whether two signals are the same or different (ie signal recognition). For example, when signalers must produce a signal repeatedly (such as an identity signature), higher magnitude signals may be beneficial because slight variations are less perceptible, which may aid consistency and recognizability. However, this would come at the cost of making the signal less distinctive compared to other identity signatures, or forgeries of that signature (Caves et al. 2021; Dixit et al. 2023), and therefore there may be both benefits and costs to high magnitude identity signatures. Overall, our finding that the magnitude effect is common suggests that it may have the potential to affect signal evolution across a wide range of different systems.

If processing stimuli according to Weber’s Law makes it difficult to distinguish stimuli with high magnitudes, which could be costly for receivers, why is this found on average? It has been suggested that sensory systems tend to follow Weber’s Law because it is an efficient way to process stimuli (Laughlin 1989; Portugal and Svaiter 2011), due to the fact that proportional relationships between stimuli are often important. For example, objects reflect back a constant proportion of ambient light, so the proportional contrast between objects remains the same regardless of ambient light level (Shapley and Enroth-Cugell 1984). Similarly, objects which are further away appear smaller, but two objects that are the same distance away from an observer will always have a constant proportional size difference. We found that perception conforms to Weber’s Law on average, and that this can be seen across a range of sensory modalities, which may support the hypothesis that sensory systems are often optimized to perceive proportional relationships. By contrast, the high amount of variability that we found between species and stimuli suggests that discrimination is not constrained to follow Weber’s Law, and that the strength of the magnitude effect may be able to respond to other selection pressures. Overall, Weber’s Law appears to be common, perhaps due to its efficiency, but not universal.

An important limitation of our study is that the data we analyzed were not direct measurements of sensory perception, but choices between stimuli. Failing to discriminate between two options may not necessarily reflect an inability to perceive a difference—for example, during foraging, individuals may deliberately return to previously unrewarding options as a form of exploratory behavior (Nachev et al. 2013). Choice tests therefore measure motivation as well as perception, and this motivation may depend on the fitness costs and benefits of each choice (for an example of adaptive incorrect decision-making, due to a trade-off between accuracy and speed, see Chittka et al. (2009)). Furthermore, decisions are often made by integrating several stimuli across different modalities, but to quantify whether perception follows Weber’s Law, a single axis for stimulus magnitude must be defined (Dixit et al. 2021). This means that when choosing between natural stimuli, animals may sometimes make decisions based on aspects of the stimuli that researchers have not accounted for (Caves et al. 2019). The way in which stimuli are presented might also aid or hinder comparison: for instance, ambient light conditions could affect visual discrimination ability (Avilés et al. 2011), or the volume of a sound could affect frequency discrimination (Wier et al. 1977). These factors are general problems with behavioral studies testing discrimination between stimuli, and thus ideally researchers should conduct tests of motivation, and consider all potential stimuli and stimulus modalities when studying discrimination.

Another possible limitation of our study is that perception may not always be described well by the generalized form of Weber’s Law. The generalized form of Weber’s Law used in our analysis includes a parameter, *k*, that allows the strength and direction of the magnitude effect to vary. However, this model assumes that as stimulus magnitude increases, discrimination ability either increases or decreases—a unidirectional effect. One study that was included in the meta-analysis (Bizo et al. 2006), however, reported that discrimination was best at intermediate magnitudes, with worse discrimination at both very high and very low magnitudes. Our measure of the magnitude effect does not capture such relationships, which suggests that our estimates of the magnitude effect may depend on the range of magnitudes for which discrimination was measured, which is an additional source of uncertainty in all estimates. Furthermore, if discrimination is easier close to a particular stimulus magnitude, this may indicate categorical perception (Caves et al. 2018; Zipple et al. 2019; Green et al. 2020), another type of nonlinear processing which we did not consider in this study. Future studies could investigate this possibility, or determine how Weber’s Law and categorical perception may interact.

Although our literature search generated a large number of studies on a wide taxonomic range of organisms, it could not identify all relevant tests of how organisms discriminate between stimuli of different magnitudes (see eg Basolo 1990; Zuk et al. 1990; Pryke and Andersson 2008; Johnston et al. 2023), as evidenced by the lack of overlap between the review and the database search. The studies we sought are divided across fields (including behavioral ecology, psychology, and neuroscience), and often use very general terms (such as “stimuli,” “discrimination,” and “sensory perception”) used in a variety of contexts, making it difficult to hone in on relevant papers without biasing search terms to capture particular studies. Furthermore, for many studies, the raw data were not available, making it impossible to include these data in our study. This means that our analysis was not comprehensive, and so we hope that our results inspire future research testing for the magnitude effect, perhaps within specific taxa and/or sensory modalities.

In summary, our study adds to the growing body of evidence suggesting that, although it is frequently assumed that stimuli are compared according to the absolute differences between them, this is often not the case (Akre and Johnsen 2014; Dixit et al. 2021; Bullough et al. 2023). The magnitude effect can be found across stimuli, species, and sensory modalities, which may have implications for a wide range of evolutionary processes (Akre and Johnsen 2014; Dixit et al. 2021). We found considerable variation in the size of the magnitude effect, suggesting that stimulus magnitudes may be processed differently in different contexts. Our results suggest that the potential effects of this sensory bias on signal evolution and receiver behavior are worth further consideration in a wide range of systems.

## Supplementary Material

araf025_suppl_Supplementary_Datas_S1

araf025_suppl_Supplementary_Datas_S2

## Data Availability

Analyses reported in this article can be reproduced using the data provided by Worsley et al. (2025). *kber* can be downloaded from GitHub: https://github.com/mzw22/kber.
